# Quantitative electroencephalography characteristics in delirium with various etiologies: A multicenter study

**DOI:** 10.1016/j.nicl.2025.103871

**Published:** 2025-08-18

**Authors:** Julia van der A, Robert Fleischmann, Annerose Mengel, Lisette Vernooij, Cornelis Stam, Sophie Leroy, Pauline Schneider, Johannes Ehler, Arjen Slooter, Edwin van Dellen

**Affiliations:** a Department of Intensive Care Medicine, University Medical Center Utrecht Brain Center, University Medical Center Utrecht, Utrecht University, Utrecht, the Netherlands; b Department of Psychiatry, University Medical Center Utrecht Brain Center, University Medical Center Utrecht, Utrecht University, Utrecht, the Netherlands; c Department of Neurology, University Medicine Greifswald, Greifswald, Germany; d Department of Neurology & Stroke, Hertie Institute for Clinical Brain Research, University of Tübingen, Tübingen, Germany; e Department of Clinical Neurophysiology, Amsterdam University Medical Centers, Amsterdam, the Netherlands; f Department of Anesthesiology and Intensive Care Medicine, Jena University Hospital, Jena, Germany

**Keywords:** Delirium, Delirium subtypes, Quantitative electroencephalography, EEG slowing, Neurophysiological signature

## Abstract

**Aim:**

Delirium is a manifestation of acute encephalopathy, which has a heterogeneous etiology. This study aimed to investigate whether quantitative electroencephalography (qEEG) characteristics of delirium differ among etiology-based delirium subtypes or converge on common neurophysiological patterns.

**Methods:**

In this multicenter observational study, we analyzed qEEG data from 377 patients (173 delirious, 204 non-delirious) consecutively enrolled across three sites. Etiology-based delirium subtypes included post-stroke, medical and postoperative delirium diagnosed by staff physicians based on the Diagnostic and Statistical Manual of Mental Disorders-IV or 5 criteria. We compared peak frequency, relative power, and phase lag index (PLI) between delirious and non-delirious patients across different etiologies using standardized mean differences (SMDs) with 95% confidence intervals (CI).

**Results:**

Among spectral qEEG measures, there were no differences between subgroups. The peak frequency showed a consistent decrease between all delirium groups and non-delirious controls (SMD = -0.81, CI:-1.50 to −0.13), relative delta power increased (SMD = 1.44, CI:0.61 to 2.26), and relative beta power decreased (SMD = -1.72, CI:-2.46 to −0.97). Effect sizes differences in PLI between delirious and non-delirious controls were small and not consistent across subtypes.

**Conclusions:**

Our findings demonstrate consistent patterns in spectral qEEG characteristics across delirium subtypes, suggesting a common neurophysiological pathway of global EEG slowing in delirium regardless of etiology. By contrast, differences in PLI did not converge across subtypes. Multicenter studies should harmonize data collection to disentangle the shared and distinct neurophysiological changes associated with delirium of varying etiologies, in order to advance our understanding of potentially convergent mechanisms across subtypes.

## Highlights

• Multicenter study analyzed EEG of 193 delirious and 204 non-delirious patients.
• Delirium is linked to consistent global EEG slowing across etiology-based subtypes.
• Phase lag index (PLI) differences did not converge across delirium subtypes.
• High heterogeneity in non-delirious controls emphasizes careful group selection.
• Harmonized data collection is crucial for future multicenter studies.

## 1 Introduction

Delirium, a clinical expression of acute encephalopathy, is an acute disturbance in attention, awareness, and cognition caused by another medical condition (Association, 2022, Slooter et al., 2020 May 13). It affects approximately 20–30 % of hospitalized older adults (Gibb et al., 2020 Apr 27). Delirium is associated with the risk of extended hospitalization, long-term cognitive dysfunction, dementia and elevated health care costs (Ely et al., 2001, Pandharipande et al., 2013 Oct 3, Gleason et al., 2015 Dec 1, Leslie and Inouye, 2011 Nov, Goldberg et al., 2020 Nov 1), posing a significant challenge in the context of an aging global population (U.S. Census Bureau. https://www.researchgate.net/profile/Paul-Kowal/publication/, 2016). Despite its prevalence and clinical significance, management of delirium is hindered by poor understanding of the pathophysiology of the underlying encephalopathy (Luetz et al., 2014, Harwood RH, Teale E. Where next for delirium research In: International Journal of Geriatric Psychiatry. John Wiley and Sons Ltd;, 2018).

Despite a uniform clinical presentation, delirium has a heterogeneous etiology. This creates uncertainty about whether etiological factors converge onto a common pathophysiological pathway. This etiological heterogeneity may complicate efforts to identify consistent biomarkers and develop effective treatments, as different subtypes of delirium may arise from distinct underlying mechanisms (Bowman et al., 2024 Jan 1). Recent attempts to distinguish delirium subtypes, for example based on precipitating factors (Potter et al., 2024 Feb, Girard et al., 2018 Mar 1) raise the question whether different precipitating conditions are also characterized by different underlying neurophysiological changes that require specific treatment. Alternatively, delirium may converge on a common neurophysiological pattern regardless of its heterogeneous etiology (van Montfort et al., 2019).

Electroencephalography (EEG) has become a valuable method for investigating the neurophysiological changes associated with delirium, offering non-invasive monitoring of brain activity at bedside. Quantitative EEG (qEEG) parameters, including spectral power distribution and peak frequency, as well as functional connectivity measures such as the phase lag index (PLI), have all been studied in delirious patients (Boord et al., 2021 Jan). Most studies have observed increased generalized slowing of EEG activity, including reduced alpha power (Roberson et al., 2023 Feb) and increased activity in the theta and delta bands (Fleischmann et al., 2019 Mar 1, Numan et al., 2019 Jan, Van Der Kooi et al., 2015 Jan 1), as well as decreased functional connectivity (Fleischmann et al., 2019, Numan et al., 2017 Jun, van Dellen et al., 2014 Aug 1). However, studies often used distinct outcome measures, and even among those using the same outcomes, methodological variations in EEG preprocessing may have influenced results. Moreover, most studies have treated delirium as a homogeneous clinical syndrome without stratifying by predisposing and/or precipitating factors, potentially masking neurophysiological signatures specific to etiological subtypes. To the best of our knowledge, only one study has specifically investigated whether qEEG features could differentiate between postoperative and non-postoperative delirium in single-channel EEG. They found no evidence of distinct EEG profiles between these subtypes using a machine learning approach (Lodema et al., 2024 May).

To elucidate potential neurophysiological differences between delirium subtypes, we conducted a multicenter study investigating qEEG measures in delirious patients compared to non-delirious patients across various etiologies using standardized methodology. We hypothesized that qEEG measures showed consistent differences across various types of delirium, which would support the existence of a final common pathophysiological pathway.

## 2 Materials and methods

### 2.1 Study design

This multicenter observational study analyzed delirium and EEG data from three study sites. Ethical approval was obtained from the institutional ethics review boards of the University Medicine Greifswald (#BB103/20), University of Tübingen (#621/2022BO2), and University Medical Center Utrecht (#11–073), and all procedures followed the latest version of the Declaration of Helsinki. To ensure patient privacy and confidentiality, data at each study site was stored and analyzed using pseudonyms, and only anonymized data was shared between sites. At the Greifswald and Tübingen study sites, written informed consent was obtained from non-delirious patients or from legal representatives for delirious patients, with written re-consent obtained from patients after delirium resolution. In the Utrecht study site, written informed consent was obtained preoperatively.

Patients were assigned one of three delirium types, which were post-stroke delirium (PSD), medical delirium (MED) and postoperative delirium (POD), depending on all available information on etiological factors. The MED group consisted of non-surgical, non-stroke patients experiencing delirium caused by a range of non-neurological conditions, such as metabolic or infectious disorders. These disorders could either co-exist with an underlying neurological condition (e.g., Parkinson’s disease) or arise following an initial suspicion of a neurological diagnosis. Specific precipitating factors were not further classified due to frequent overlap (e.g., sepsis resulting in renal failure). Non-delirious controls for either delirium subtype consisted of patients affected by similar conditions but without delirium. See Table 1 for an overview of number of included participants per etiology and site.

**Table 1:** Description of delirious and non-delirious patients overall and per etiology. Sites Greifswald and Tübingen had a focus on post-stroke delirium and medical delirium, while Utrecht exclusively enrolled patients after cardiac surgery. Del+ = delirious, Del- = non-delirious, SD = standard deviation, − = not applicable. † Significant difference in age between delirious and non-delirious patients (*p* < 0.001) ‡ Significant difference in age between the Del + groups (POD, PSD, and MED), as indicated by a Kruskal-Wallis H test, χ^2^ (2) = 10.41, *p* = 0.0055. Post-hoc analysis (Dunn's test with Bonferroni adjustment) revealed a significant age difference specifically between the POD and PSD groups (*p* = 0.0065), but not between PSD and MED, or MED and POD.

			Etiology					
	Overall		Post-stroke		Medical		Postoperative	
	Del+	Del-	Del+	Del-	Del+	Del-	Del+	Del-
**Sample size,***n*	173	204	82	113	67	68	24	23
**Age,** mean ± SD	79.2 ± 9.2†	72.9 ± 13.1†	80.8 ± 8.5‡	73.4 ± 10.8	78.1 ± 10.5	71.2 ± 17.2	75.9 ± 5.8‡	75.7 ± 6.9
**Sex,***male/female*	93 / 80	113 / 91	41 / 41	67 / 46	40 / 27	32 / 36	12 / 12	14 / 9
**Study sites,***n*								
*Greifswald*	79	111	38	70	41	41	−	−
*Tübingen*	70	70	44	43	26	27	−	−
*Utrecht*	24	23	−	−	−	−	24	23

At the Greifswald study site, patients were enrolled from the stroke unit and general neurological ward. Patients from the general neurological ward comprised two groups: (Association, 2022) those with pre-existing neurological conditions (e.g., Parkinson's disease) who developed medical conditions (e.g., renal failure), and (Slooter et al., 2020 May 13) those initially suspected of neurological conditions but ultimately diagnosed with purely medical conditions without underlying neurological disease. EEG data were acquired during clinical routine for patients demonstrating changes in cognitive function, suggestive of delirium, to rule out non-convulsive epileptic activity. Delirium assessment was conducted using the Confusion Assessment Method (CAM), developed for patients in general medicine wards, and validated for use in stroke patients (Fleischmann et al., 2021 Feb 1, Inouye et al., 1990 Dec 15). An experienced staff physician confirmed the presence of delirium using the DSM, 5th edition (DSM-5) criteria. The study also included non-delirious control patients, comprising patients initially evaluated for epilepsy but ultimately diagnosed with non-neurological conditions such as syncope, as well as patients with stroke but without delirium.

At the Tübingen study site, patients from the neurological ICU and stroke unit were enrolled. Similar to Greifswald, EEG data were acquired during clinical routine for patients demonstrating changes in cognitive function, suggestive of delirium, to exclude the possibility of non-convulsive epileptic activity. Delirium assessment was performed using the Intensive Care Delirium Screening Checklist (ICDSC), which has been validated for use in stroke patients (Boßelmann et al., 2019 Nov). An experienced staff physician confirmed the presence of delirium using DSM-5 criteria. The non-delirious control patients were evaluated for the same reasons as delirious patients but were ultimately not diagnosed with delirium or encephalopathy.

At the Utrecht study site, EEG data from cardiac surgery patients with and without delirium was collected as previously described (Numan et al., 2017 Jun). Patients were recruited preoperatively and assessed for delirium during the first five consecutive days after cardiac surgery. Researchers conducted a chart review and used the confusion assessment method for intensive care unit (ICU) patients (CAM-ICU) (Ely EW, Inouye SK, Bernard GR, Gordon S, Francis J, May L, et al. Delirium in Mechanically Ventilated Patients Validity and Reliability of the Confusion Assessment Method for the Intensive Care Unit (CAM-ICU) [Internet]., 2001). After initial assessment, a geriatrician, neurologist, or psychiatrist classified patients as delirious or non-delirious based on the Diagnostic and Statistical Manual of Mental Disorders (DSM) fourth edition, text revision criteria (DSM-IV-TR). Non-delirious control patients were matched on age, sex and type of surgery on group level.

### 2.2 EEG acquisition and preprocessing

Sites used different systems for EEG data registration: Greifswald (waveguard connect® and eego® amplifier, ANT Neuro, Enschede, Netherlands), Tübingen (waveguard connect® and xltek EEG v32 amplifier, Natus Medical Incorporated, Middleton, WI, USA), Utrecht (Micromed, Treviso, Italy). All sites used EEG systems with 21 channels with a 512 Hz sampling rate and adhered to the standard 10–20 electrode placement system, in accordance with International Federation of Clinical Neurophysiology (IFCN) guidelines (Nuwer et al., 1998 Mar). At Greifswald and Tübingen, electrodes A1 and A2 were systematically excluded. Other electrodes were excluded if they contained artifacts leading to an insufficient number of artifact-free epochs. This generally applied for electrodes Fp1 and Fp2, leaving 17–19 electrodes for analyses. At Utrecht, electrodes A1, A2, Fp1, and Fp2 were systematically excluded from analysis due to consistent eye movement and muscle artifacts, leaving 17 channels for analysis. No channel interpolation was performed at any site.

The Greifswald and Tübingen study sites imported data into MATLAB and used the FieldTrip toolbox for visualization and manual artifact rejection (Oostenveld et al., 2011). The Utrecht site manually selected epochs, excluding eye movement and muscle artifacts. Greifswald and Tübingen both included 10 artifact free epochs of 8 s with eyes closed for further analysis. If more than 10 epochs were available, epochs were randomly chosen from all available epochs. Utrecht included the first 4 artifact-free epochs of 8 s with eyes closed for further analysis due to data quality constraints that limited the availability of additional clean epochs. However, prior stability analysis has demonstrated that four epochs of 8 s is sufficient for stable results (van Dellen et al., 2014 Aug 1).

### 2.3 Calculation of qEEG measures

EEG processing was performed using Brainwave (*Version 0.9.165.57, available on* https://github.com/CornelisStam/BrainWave). Data was referenced to an average reference montage, and relative spectral power and peak frequency (between 4 and 13 Hz) were computed using a Fast Fourier Transform, first for each channel and then averaged across all channels to obtain a single value per participant. Relative power was calculated by dividing the absolute power per frequency band by the total power of the delta (0.5–4 Hz), theta (4–8 Hz), alpha (8–13 Hz) and lower beta (13–20 Hz) frequency bands. To minimize the influence of muscle activity, only the lower beta frequencies (13–20 Hz) were included (Whitham et al., 2007 Aug). The mean phase lag index (PLI) was used to calculate the functional connectivity strength (Stam et al., 2007 Nov). The PLI is based on the Hilbert transformed instantaneous phase differences, capturing the asymmetry in the distribution of phase leading and lagging between two signals. PLI values range between 0–1; 1 indicates complete phase locking and a PLI of 0 means no phase synchronization. The average overall PLI values between all available channel pairs was calculated per epoch and averaged to a mean PLI per frequency band per subject.

### 2.4 Data handling and statistical analyses

All statistical analyses were performed in RStudio version 4.2.2 (R Core Team, 2021). Clinical and demographic characteristics of study participants were described using means and standard deviations (SD) for continuous variables and frequencies for categorical variables. Clinical and demographic group-related differences between delirious and non-delirious patients were analyzed using a Mann-Whitney *U* test for age and a chi-square test for sex.

qEEG characteristics were compared between patients with and without delirium across various etiologies. To account for center effects, analyses were performed separately for each etiology and study site, resulting in five subgroups. For each qEEG characteristic, means and corresponding 95 % confidence intervals (CIs) were calculated for each subset using bootstrapping (1000 iterations) because data did not follow a Gaussian distribution. To quantify the magnitude of the differences between subsets, standardized mean differences (SMDs) were calculated using Hedges' g, which provides a bias-corrected estimate. The pooled SMD was calculated as a weighted average of the individual subgroup SMDs, ensuring that subgroups with larger sample sizes and smaller variances contributed more to the pooled estimate. Weights for each subgroup were determined inversely proportional to the sum of the within-study variance and the estimated between-study variance (tau2). The between-study variance was estimated using the restricted maximum likelihood (REML) method. The I2 statistic was calculated for each qEEG characteristic and separately for patients with and without delirium to investigate whether variance in qEEG characteristics was primarily present in either group. To assess the potential influence of age on the primary analysis, we performed separate linear regression analyses with each qEEG characteristic as the dependent variable, delirium status as independent variable with age as covariate. Additionally, to evaluate the influence of site-differences in recruitment, department, and number of epochs, a sensitivity analysis excluding data originating from the Utrecht site was conducted.

## 3 Results

### 3.1 Study population characteristics

A total of 377 patients were included in the study (mean age 75.8 years, SD 11.9; 55 % male). Of these, 173 patients were classified as delirious, while 204 were classified as non-delirious. Patients with delirium (mean age 79.2 years, SD 9.2) were older (*p* < 0.001) compared to non-delirious patients (mean age 72.9 years, SD 13.1), there was no significant difference (*p* = 0.84) in the male-to-female ratio between the groups (0.86 and 0.81, respectively). The delirium subtypes were categorized as follows: PSD (n = 82), MED (n = 67), and POD (n = 24). Table 1 provides an overview of total sample demographics, and details for each delirium subtype.

### 3.2 Peak frequency

**Fig. 1:**
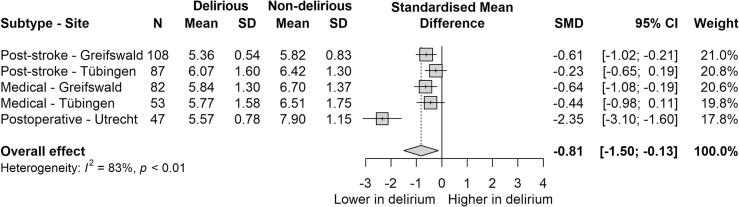
Forest plot of standardized effect sizes (*g*) of peak frequency in patients with and without delirium. Total standardized mean difference with 95 % confidence interval, weight and heterogeneity reported. SD = standard deviation, CI = confidence interval.

**Fig. 2:**
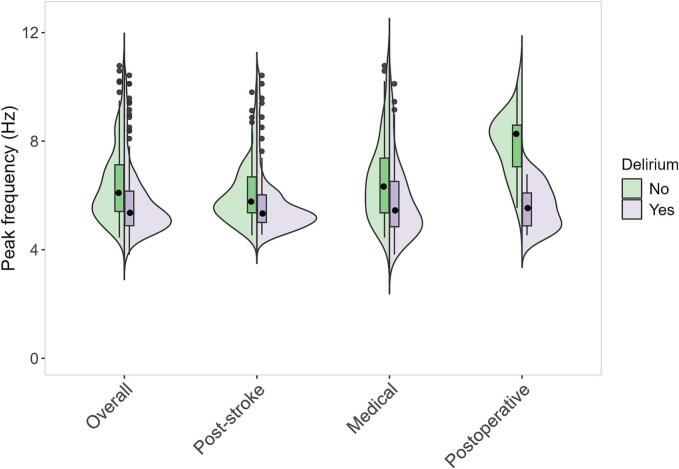
Violin plot indicating peak frequency distributions for patients with and without delirium (overall) and per different underlying etiology: post-stroke, medical and postoperative. The violin plots show the distribution of peak frequencies, with box plots indicating median and interquartile ranges. Green represents patients without delirium, while purple represents those with delirium. Hz = Hertz.

Peak frequency was consistently lower in patients with delirium compared to patients without, overall as well as across all delirium subtypes, indicated by negative standardized mean differences (SMDs) for all subgroups (Fig. 1, Fig. 2). The pooled random effects SMD was −0.81 (95 % CI: −1.50 to −0.13) indicating a significant moderate to large effect size. The largest effect was observed in the POD subgroup (SMD = −2.35, 95 % CI: −3.10 to −1.60). Among subgroups we observed I^2^ = 83 % (p < 0.01) indicating substantial variability in the magnitude of this effect across subgroups.

### 3.3 Spectral power

**Fig. 3:**
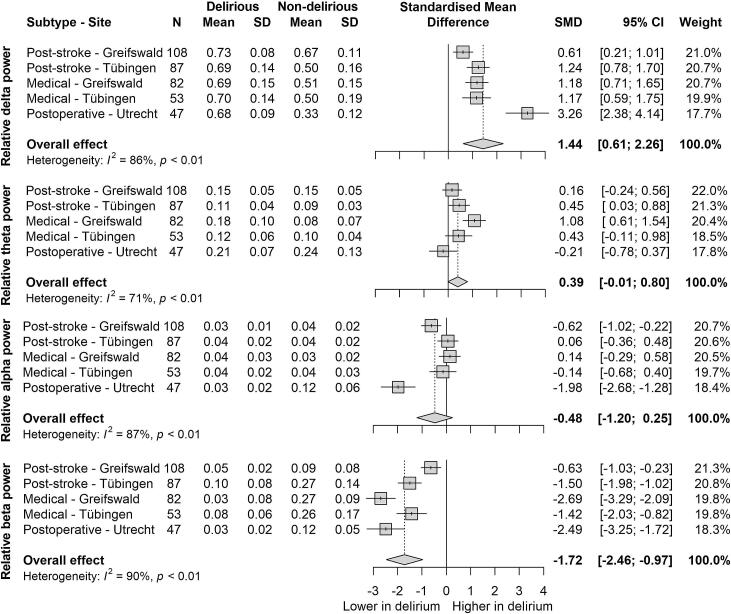
Forest plot of standardized effect sizes (*g*) of relative power per frequency band (delta, theta, alpha, beta) in patients with and without delirium. Total standardized mean difference with 95 % confidence interval, weight and heterogeneity reported. SD = standard deviation, CI = confidence interval.

**Fig. 4:**
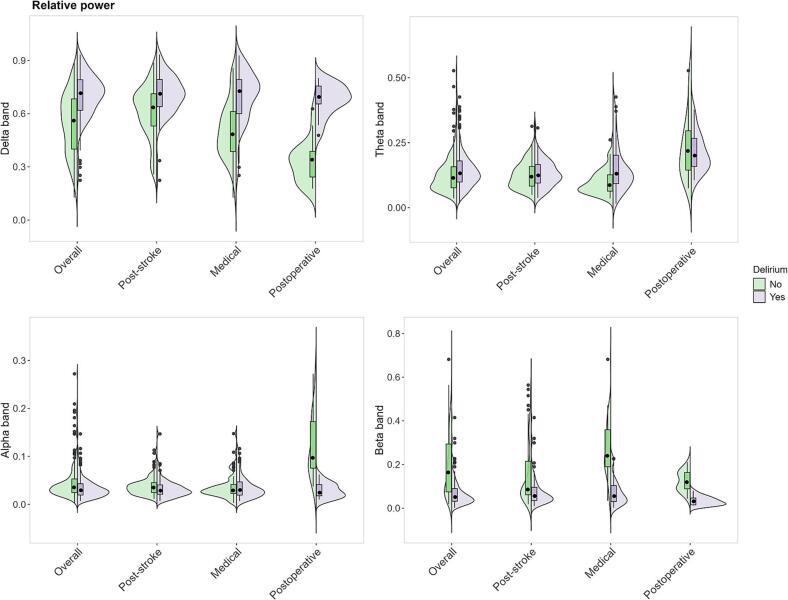
Violin plot indicating relative power per frequency band (delta, theta, alpha, beta) distributions for patients with and without delirium (overall) and per different underlying etiology: post-stroke, medical and postoperative. The violin plots show the distribution of relative power with box plots indicating median and interquartile ranges. Green represents patients without delirium, while purple represents those with delirium.

Relative delta power was consistently higher in delirious patients compared to non-delirious patients overall, as well as across all subgroups, with a large overall effect (SMD = 1.44, 95 % CI: 0.61 to 2.26, Fig. 3, Fig. 4). The effect was most pronounced in POD (SMD = 3.26, 95 % CI: 2.38 to 4.14). Relative theta power was higher in delirious patients compared to non-delirious patients in most subgroups, resulting in a small overall effect (SMD = 0.39, 95 % CI: −0.01 to 0.80). However, in a sensitivity analysis excluding the Utrecht study site, this effect became statistically significant (SMD = 0.52, 95 % CI: 0.13 to 0.91; Supplementary Material S1). Relative alpha power showed a trend towards being lower in delirious patients, but the overall effect was not statistically significant (SMD = −0.48, 95 % CI: −1.20 to 0.25). Relative beta power demonstrated the most consistent and pronounced decrease in delirious patients (SMD = −1.72, 95 % CI: −2.46 to −0.97). All delirium types showed decreased beta power compared to non-delirious controls, with MED patients from Greifswald exhibiting the largest decrease (SMD = −2.69, 95 % CI: −3.29 to −2.09). High heterogeneity was observed across all frequency bands (I^2^ ranging from 71 % to 90 %, all *p* < 0.01).

### 3.4 Phase lag index

**Fig. 5:**
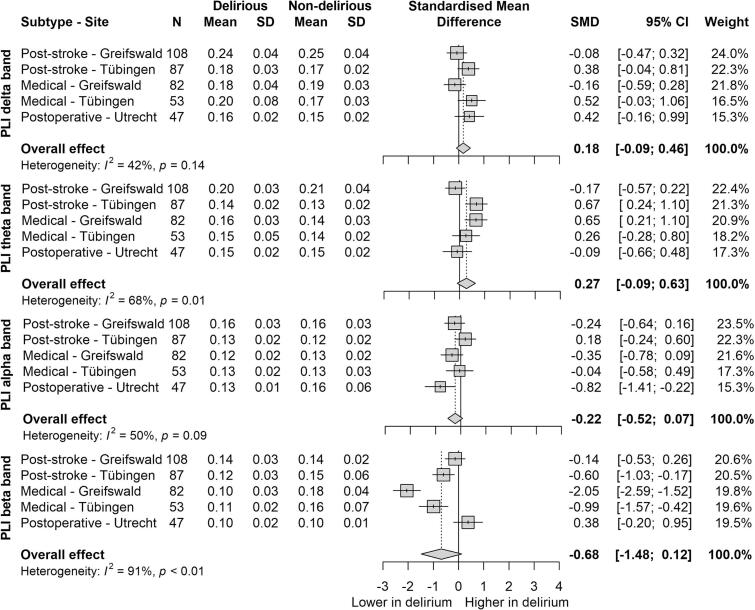
Forest plot of standardized effect sizes (*g*) of phase lag index (PLI) per frequency band (delta, theta, alpha, beta) in patients with and without delirium. Total standardized mean difference with 95 % confidence interval, weight and heterogeneity reported. SD = standard deviation, CI = confidence interval.

**Fig. 6:**
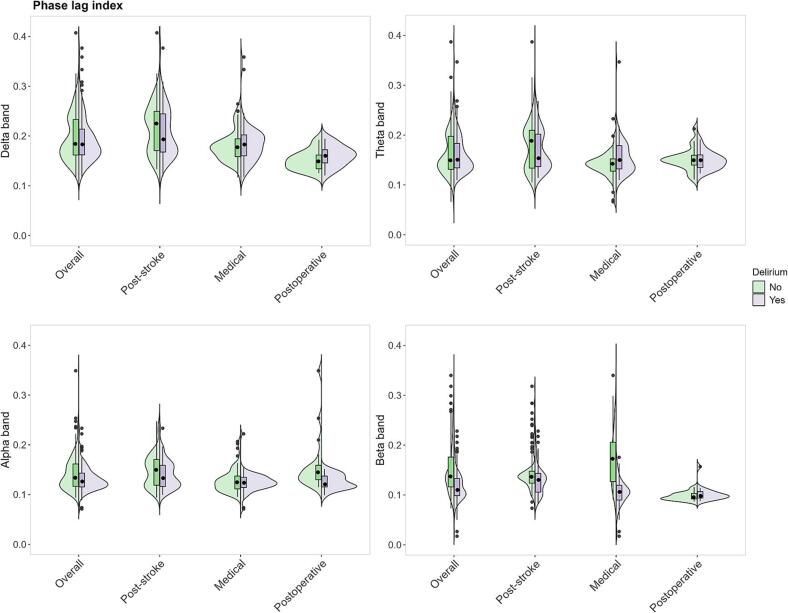
Violin plot indicating phase lag index (PLI) distributions per frequency band (delta, theta, alpha, beta) for patients with and without delirium (overall) and per different underlying etiology: post-stroke, medical and postoperative. The violin plots show the distribution of peak frequencies, with box plots indicating median and interquartile ranges. Green represents patients without delirium, while purple represents those with delirium.

Some individual study sites and subtypes showed significant differences, with PSD patients from Tubingen showing increased PLI in the theta band (SMD = 0.67, 95 % CI: 0.24 to 1.10) and MED patients from Greifswald showing decreased PLI in the beta band (SMD = −2.05, 95 % CI: −2.59 to −1.52). However, when pooled across all sites and subtypes, no significant differences were observed for the PLI across frequency bands in patients with and without delirium (Fig. 5, Fig. 6). There was a trend towards an increased PLI in the delta (SMD = 0.18, 95 % CI: −0.09 to 0.46) and theta bands (SMD = 0.27, 95 % CI: −0.09 to 0.63), and a trend for decreased PLI in the alpha (SMD = −0.22, 95 % CI: −0.52 to 0.07) and beta bands (SMD = −0.68, 95 % CI: −1.48 to 0.12) in delirious patients. Notably, a sensitivity analysis excluding the Utrecht study site revealed a significant decrease in the beta band PLI (SMD = −0.93, 95 % CI: −1.73 to −0.13; Supplementary Material S1). Heterogeneity was moderate in the delta and alpha band PLI (I^2^ = 42 %, *p* = 0.14; I^2^ = 50 %, *p* = 0.09), suggesting some consistency in the small effect size across subgroups, whereas PLI in the theta and beta frequency bands showed substantial (I^2^ = 68 %, p = 0.01) to high heterogeneity (I^2^ = 91 %, p < 0.01), respectively.

### 3.5 Heterogeneity between delirious and non-delirious patients

Heterogeneity analysis using I^2^ statistics revealed distinct patterns between patients with and without delirium regarding various qEEG characteristics (Supplementary Table S2). Non-delirious patients consistently showed high heterogeneity (I^2^ > 0.90, p < 0.001) for all qEEG characteristics. In contrast, heterogeneity in delirious patients varied across qEEG characteristics. Peak frequency, relative delta and alpha power demonstrated lower heterogeneity in delirious patients (I^2^ = 0.656, p = 0.020; I^2^ = 0.345, p = 0.191; I^2^ = 0.360, p = 0.181, respectively) compared to non-delirious patients. This suggests more consistent qEEG patterns among delirious patients compared to non-delirious controls.

### 3.6 Effect of age on qEEG characteristics

As patients with delirium were on average older compared to patients without delirium, we performed a sensitivity analysis to assess the potential influence of age on our primary analysis. Regression models with qEEG characteristics as dependent variables, delirium status as independent variable and age as covariate indicated that age was not a significant confounder in any of the models (all *p* > 0.05, Supplementary Table S3).

## 4 Discussion

In summary, this multicenter study investigated qEEG characteristics in patients with delirium of various etiologies compared to non-delirious patients. Overall, we found that delirium was associated with a decrease in peak frequency, an increase in relative delta power, and a decrease in relative beta power. The PLI showed no consistent differences between delirious and non-delirious patients across subgroups for any of the frequency bands. Additionally, we observed larger heterogeneity regarding various qEEG characteristics in non-delirious patients compared to delirious patients.

Our findings reveal a consistency in spectral EEG characteristics across various delirium types, challenging the notion that delirium due to different etiologies (e.g., PSD, MED, POD) represent fundamentally distinct underlying substrates. This consistency is particularly noteworthy in post-stroke patients. While stroke itself is known to alter the balance of excitatory and inhibitory neuronal activity, leading to local EEG slowing including reduced alpha power and increased delta and theta band power (Bentes et al., 2018 Aug 1, Finnigan et al., 2016 Feb 1, Finnigan and van Putten, 2013, Sheorajpanday et al., 2011), our findings reveal an additional global EEG slowing effect in PSD compared to non-delirious post-stroke patients. Our observations align with findings by Mintz et al (2024), who observed that stroke patients had more generalized EEG slowing on days with delirium (Mintz et al., 2024). These differences in spectral EEG characteristics, even in patients with structural brain lesions who have often been excluded from EEG-based delirium research, suggests a common neurophysiological pattern of global EEG slowing underlying delirium regardless of its precipitating cause.

While the overall patterns of relative EEG power were consistent, the magnitude of these differences varied across subgroups. The POD subgroup showed significantly larger effect sizes in peak frequency, relative delta, and alpha power than other subgroups. This variability may reflect differences in the non-delirious patients used for comparison, as our analysis revealed lower qEEG heterogeneity among delirious patients compared to non-delirious controls. Several factors could account for this. First, some patients may have experienced delirium that clinically resolved by the time of the EEG recording, resulting in classification in the control group, yet still displaying qEEG alterations (Boord et al., 2021 Jan). Second, control patients may have had subsyndromal delirium, which is associated with qEEG changes without manifesting the full clinical phenotype (Ditzel et al., 2022). Third, given the high prevalence of undiagnosed delirium, we cannot exclude the possibility that some patients classified as non-delirious may have, in fact, experienced delirium (Lange et al., 2019 Jul 1). It is noteworthy that while delirium assessment tools used in the current study demonstrate excellent performance in research settings (Neto et al., 2012), their sensitivity is lower in routine clinical practice (Van Eijk et al., 2011 Aug 1). This might have contributed to the observed variability in effect sizes between the postoperative subgroup and the other subgroups and underscores the importance of standardized control group selection.

The differences in PLI between delirious and non-delirious patients were generally small and did not show consistent patterns across delirium types. While these PLI differences were subtle, we observed a trend wherein changes in relative power in delirious patients corresponded to similar directional changes in PLI within the same frequency bands, which aligns with previous research (Boord et al., 2021 Jan). Although the brain network dysconnectivity hypothesis of delirium is widely recognized (van Montfort et al., 2019, Sanders, 2011 Jul, Young, 2017 Jul, Wilson et al., 2020 Nov 12), few studies have investigated PLI differences during delirium. It is plausible that risk factors contributing to the development of delirium may already influence functional connectivity, leading to minimal discernible PLI differences between delirious and non-delirious patients (van Montfort et al., 2019). Specifically, stroke may have caused network disconnections related to diaschisis, i.e. disruptions in hubs connected to the lesion site, thereby obscuring the effects on global connectivity as a potential substrate for delirium (Juhász et al., 1997). Future studies are needed to explore the impact of delirium on phase-based functional connectivity, including measures prior to the onset of delirium.

The study has several strengths. First, the multicenter design with a large number of participants and allows generalizability of our findings. Second, qEEG data were evaluated using rigorous and standardized methodology including consistency in epoch length and calculation of qEEG parameters. This approach aligns with recent calls for harmonization in delirium research, such as those made by the International Delirium Pathophysiology & Electrophysiology Network (iDEPEND) (Sanders RD, Watne L, Roberson SW, Kimchi EY, Slooter AJC, Cunningham C, et al. International Delirium Pathophysiology Electrophysiology Network for Data sharing (iDEPEND). Vol. 11, BJA Open. Elsevier B.V.;, 2024). However, several limitations should be noted. Different recruitment strategies, EEG registration systems, number of epochs and delirium detection tools were used across sites, and data on patient characteristics were limited to a small core set of variables consistently available across all sites. Furthermore, non-delirious patients were on average younger compared to delirious patients. Although our sensitivity analysis did not indicate that age significantly influenced our results, the large heterogeneity observed in non-delirious patients highlights the importance of careful control group selection for future delirium EEG studies. The sensitivity analysis excluding the Utrecht site demonstrated that methodological variations between sites might have masked significant theta power and beta PLI effects, emphasizing the need for harmonized data collection in future multicenter research.

Future multicenter studies should harmonize data collection methods prior to study initiation to ensure greater consistency. Ideally, data on delirium severity and duration, as well as medication use at the time of EEG recording, should be included. Although the current study represents an important first step towards this goal of standardization in multicenter delirium research, these improvements in data harmonization would further advance the field.

In conclusion, this multicenter study found that spectral EEG characteristics were consistent across delirium subtypes, suggesting a common neurophysiological pattern of global EEG slowing in postoperative, post-stroke and medical delirium. By contrast, differences in PLI did not converge across delirium subtypes. Multicenter approaches that harmonize data collection are essential for improving our understanding of the neurophysiological basis of delirium and its subtypes.

## CRediT authorship contribution statement

**Julia van der A:** Writing – review & editing, Writing – original draft, Visualization, Project administration, Methodology, Formal analysis, Data curation. **Robert Fleischmann:** Writing – review & editing, Writing – original draft, Project administration, Methodology, Formal analysis, Data curation, Conceptualization. **Annerose Mengel:** Writing – review & editing, Data curation. **Lisette Vernooij:** Writing – review & editing, Methodology, Formal analysis. **Cornelis Stam:** Writing – review & editing, Software, Methodology, Conceptualization. **Sophie Leroy:** Writing – review & editing, Data curation. **Pauline Schneider:** Writing – review & editing, Data curation. **Johannes Ehler:** Writing – review & editing, Methodology, Conceptualization. **Arjen Slooter:** Writing – review & editing, Supervision, Resources, Methodology, Funding acquisition, Conceptualization. **Edwin van Dellen:** Writing – review & editing, Supervision, Methodology, Formal analysis, Data curation, Conceptualization.

## Declaration of Competing Interest

The authors declare that they have no known competing financial interests or personal relationships that could have appeared to influence the work reported in this paper.

## Data Availability

Data will be made available on request.
