# Supplementary material for: Quantitative electroencephalography characteristics in delirium with various etiologies: A multicenter study
Source: Neuroimage Clin. 2025 Aug 18;48:103871. doi: 10.1016/j.nicl.2025.103871 (PMC12395080; doi:10.1016/j.nicl.2025.103871)
Supplement: Supplementary Data 1 [file mmc1.docx]

**Supplementary material**

**Quantitative electroencephalography characteristics in delirium with various etiologies: A multicenter study**

Julia van der A, MSc, Robert Fleischmann, MD, PhD, Annerose Mengel MD, PhD, Lisette Vernooij, PhD, Cornelis Stam, MD, PhD, Sophie Leroy, MD, Pauline Schneider, Johannes Ehler, MD, PhD, Arjen Slooter, MD, PhD Edwin van Dellen, MD, PhD

**Supplementary material S1. Sensitivity analysis without Utrecht cohort to investigate site-specific methodological influences**


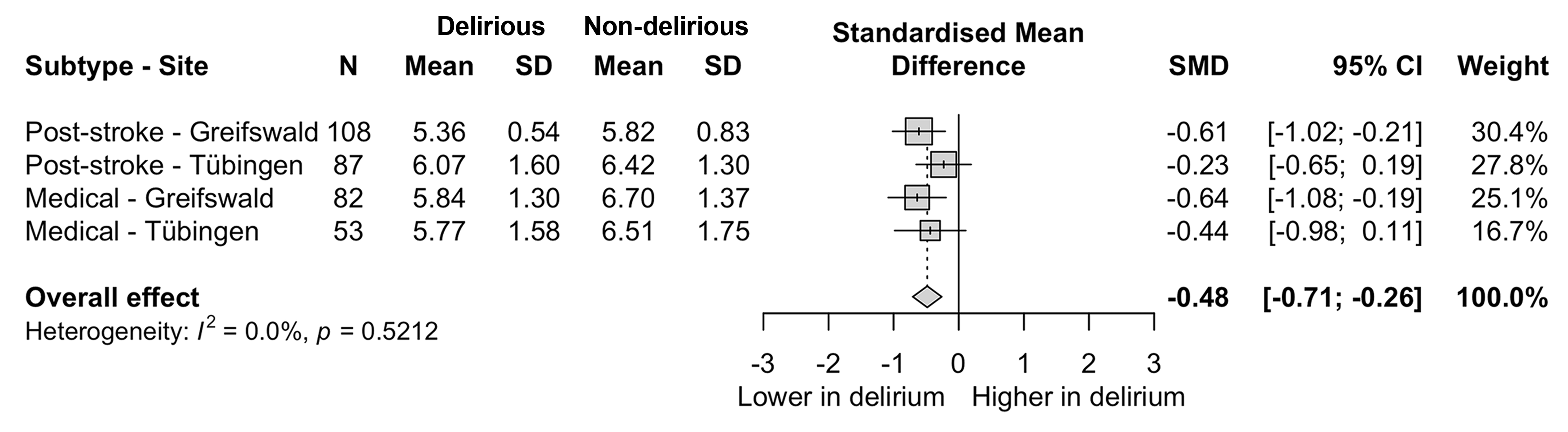


**Supplementary Figure 1.1** Forest plot of standardized effect sizes (*g*) of peak frequency in patients with and without delirium. Total standardized mean difference with 95% confidence interval, weight and heterogeneity reported. SD = standard deviation, CI = confidence interval.


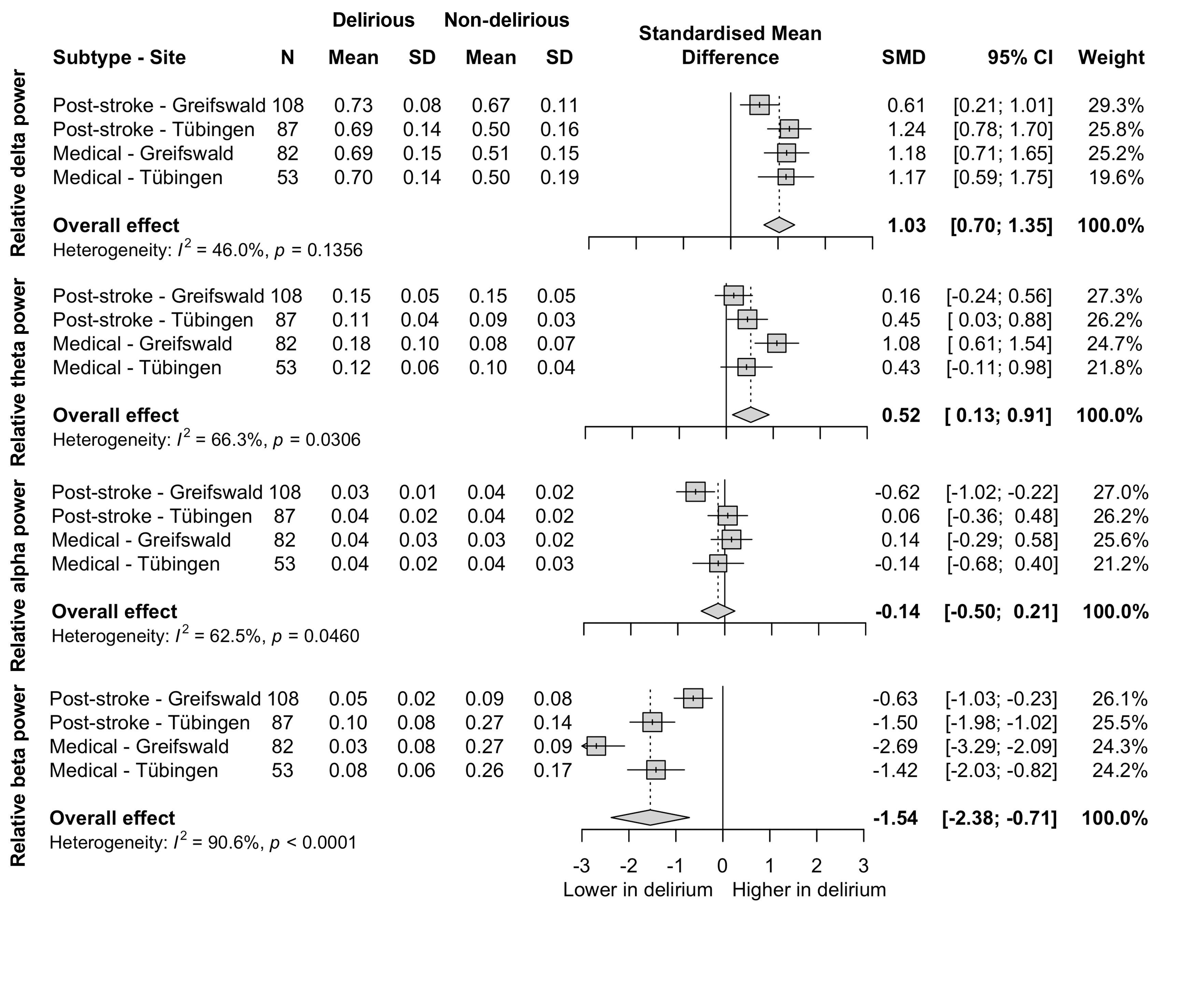


**Supplementary Figure 1.2** Forest plot of standardized effect sizes (*g*) of relative power per frequency band (delta, theta, alpha, beta) in patients with and without delirium. Total standardized mean difference with 95% confidence interval, weight and heterogeneity reported. SD = standard deviation, CI = confidence interval.


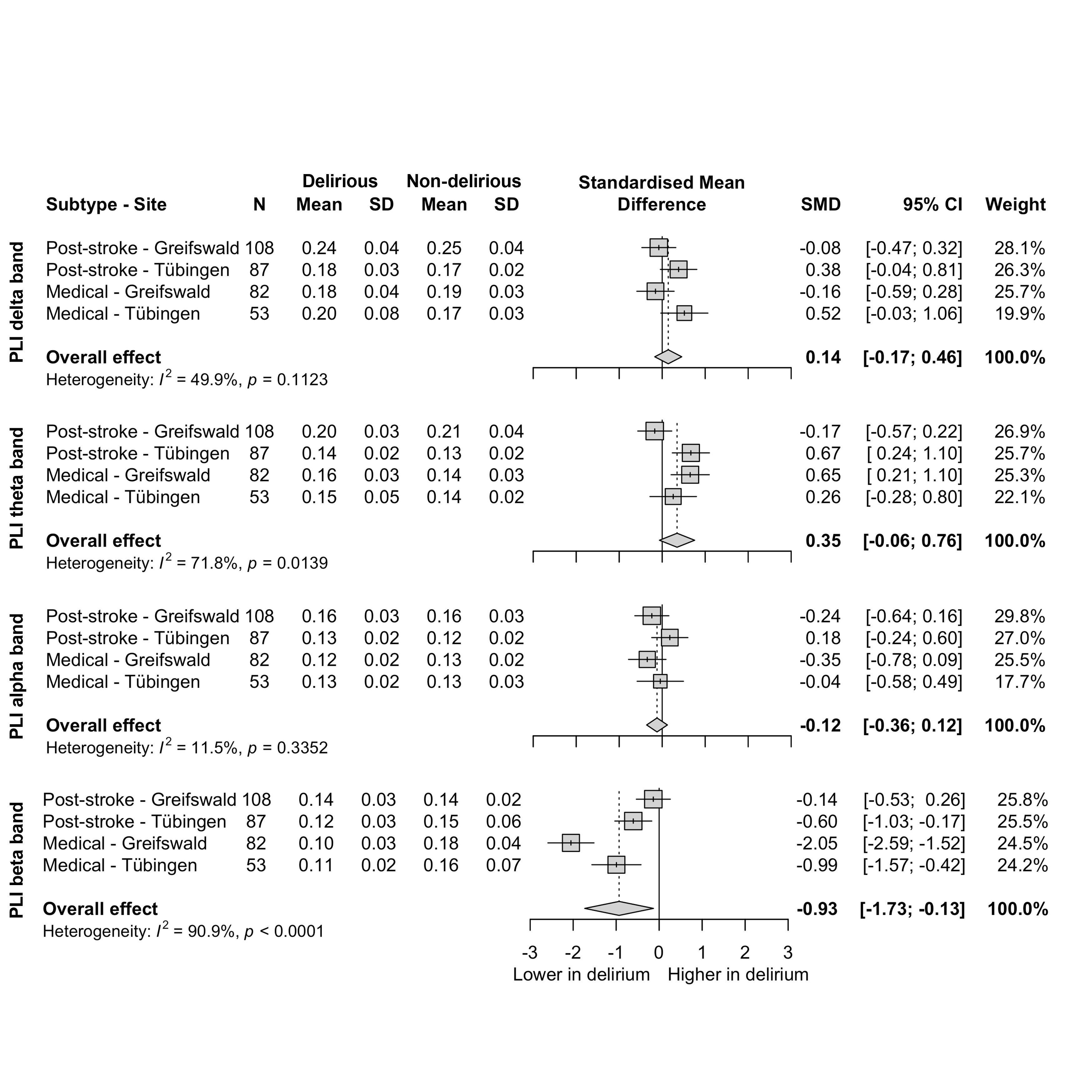


**Supplementary Figure 1.3** Forest plot of standardized effect sizes (*g*) of phase lag index (PLI) per frequency band (delta, theta, alpha, beta) in patients with and without delirium. Total standardized mean difference with 95% confidence interval, weight and heterogeneity reported. SD = standard deviation, CI = confidence interval.

**Supplementary table S2. I^2^ statistic calculated per EEG characteristic for patients with and without delirium separately**

|  | **Delirious** | | **Non-delirious** | |
| --- | --- | --- | --- | --- |
|  | *I^2^* | *p*-value | *I^2^* | *p*-value |
| Peak frequency | 0.656 | 0.020* | 0.945 | < 0.001* |
| Relative delta power | 0.345 | 0.191 | 0.976 | < 0.001* |
| Relative theta power | 0.926 | < 0.001* | 0.950 | < 0.001* |
| Relative alpha power | 0.360 | 0.181 | 0.908 | < 0.001* |
| Relative beta power | 0.889 | < 0.001* | 0.976 | < 0.001* |
| PLI delta | 0.962 | < 0.001* | 0.986 | < 0.001* |
| PLI theta | 0.959 | < 0.001* | 0.982 | < 0.001* |
| PLI alpha | 0.917 | < 0.001* | 0.956 | < 0.001* |
| PLI beta | 0.920 | < 0.001* | 0.986 | < 0.001* |

PLI = Phase lag index, * Significant at *p* < 0.05.

**Supplementary table S3. Impact of adjustment for age in the association of delirium with quantitative electroencephalography (qEEG) characteristics**

|  | **Not adjusted^†^** | |  | **Adjusted for age^‡^** | | |  |
| --- | --- | --- | --- | --- | --- | --- | --- |
|  | *β* | *p*-value |  | *β* | *p*-value | *p*-value for age | % change in *β* |
| Peak frequency | -0.667 | <0.001 |  | -0.732 | <0.001 | 0.091 | 9.66 |
| Relative delta power | 0.153 | <0.001 |  | 0.154 | <0.001 | 0.905 | 0.33 |
| Relative theta power | 0.026 | 0.002 |  | 0.026 | 0.003 | 0.831 | -1.85 |
| Relative alpha power | -0.012 | <0.001 |  | -0.013 | <0.001 | 0.322 | 7.87 |
| Relative beta power | -0.129 | <0.001 |  | -0.126 | <0.001 | 0.363 | -2.28 |
| PLI delta | -0.003 | 0.588 |  | -0.003 | 0.590 | 0.947 | 3.51 |
| PLI theta | -0.001 | 0.778 |  | -0.002 | 0.665 | 0.541 | 58.87 |
| PLI alpha | -0.011 | 0.001 |  | -0.011 | 0.001 | 0.634 | 3.95 |
| PLI beta | -0.033 | <0.001 |  | -0.035 | <0.001 | 0.196 | 4.76 |

† β-coefficient and corresponding p-value of delirium status in the linear regression with qEEG characteristic as dependent variable and delirium status (yes/no) as independent variable.
‡ β-coefficient and corresponding p-value of delirium status in the linear regression analysis with qEEG characteristic as dependent variable and delirium status (yes/no) as independent variable, with adjustment for age. PLI = Phase lag index. Although adjusting for age substantially changed the estimated effect of delirium status on PLI theta, this finding is not meaningful since the effect of delirium status on PLI theta did not reach statistical significance.
